# High frequency of the IVS2-2A>G DNA sequence variation in *SLC26A5*, encoding the cochlear motor protein prestin, precludes its involvement in hereditary hearing loss

**DOI:** 10.1186/1471-2350-6-30

**Published:** 2005-08-08

**Authors:** Hsiao-Yuan Tang, Anping Xia, John S Oghalai, Fred A Pereira, Raye L Alford

**Affiliations:** 1 The Bobby R. Alford Department of Otorhinolaryngology and Communicative Sciences, Baylor College of Medicine, One Baylor Plaza, NA102, Houston, TX 77030, USA; 2 Huffington Center on Aging and Department of Molecular and Cellular Biology, Baylor College of Medicine, One Baylor Plaza, N710, Houston, TX 77030, USA

## Abstract

**Background:**

Cochlear outer hair cells change their length in response to variations in membrane potential. This capability, called electromotility, is believed to enable the sensitivity and frequency selectivity of the mammalian cochlea. Prestin is a transmembrane protein required for electromotility. Homozygous prestin knockout mice are profoundly hearing impaired. In humans, a single nucleotide change in *SLC26A5*, encoding prestin, has been reported in association with hearing loss. This DNA sequence variation, IVS2-2A>G, occurs in the exon 3 splice acceptor site and is expected to abolish splicing of exon 3.

**Methods:**

To further explore the relationship between hearing loss and the IVS2-2A>G transition, and assess allele frequency, genomic DNA from hearing impaired and control subjects was analyzed by DNA sequencing. *SLC26A5 *genomic DNA sequences from human, chimp, rat, mouse, zebrafish and fruit fly were aligned and compared for evolutionary conservation of the exon 3 splice acceptor site. Alternative splice acceptor sites within intron 2 of human *SLC26A5 *were sought using a splice site prediction program from the Berkeley Drosophila Genome Project.

**Results:**

The IVS2-2A>G variant was found in a heterozygous state in 4 of 74 hearing impaired subjects of Hispanic, Caucasian or uncertain ethnicity and 4 of 150 Hispanic or Caucasian controls (p = 0.45). The IVS2-2A>G variant was not found in 106 subjects of Asian or African American descent. No homozygous subjects were identified (n = 330). Sequence alignment of *SLC26A5 *orthologs demonstrated that the A nucleotide at position IVS2-2 is invariant among several eukaryotic species. Sequence analysis also revealed five potential alternative splice acceptor sites in intron 2 of human *SLC26A5*.

**Conclusion:**

These data suggest that the IVS2-2A>G variant may not occur more frequently in hearing impaired subjects than in controls. The identification of five potential alternative splice acceptor sites in intron 2 of human *SLC26A5 *suggests a potential mechanism by which expression of prestin might be maintained in cells carrying the *SLC26A5 *IVS2-2A>G DNA sequence variation. Additional studies are needed to evaluate the effect of the IVS2-2A>G transition on splicing of *SLC26A5 *transcripts and characterize the hearing status of individuals homozygous for the IVS2-2A>G variant.

## Background

Outer hair cells (OHCs) are sensory cells of the mammalian cochlea. These cells are cylindrical in shape, with stereocilia projecting from their apical surfaces and neuronal synapses associated with their basal surfaces. Mechanical deflection of OHC stereocilia, in response to sound pressure waves, results in variations in the OHC membrane potential that trigger somatic cell length changes in synchrony with the sound wave. Hyperpolarizing potentials elongate the cell and depolarizing potentials shorten the cell. Known as electromotility, this capability is believed to amplify cochlear vibrations and enable the acute hearing sensitivity and frequency selectivity of the mammalian cochlea [1-3].

Prestin is a multipass transmembrane protein of the OHC required for electromotility [2,3]. Developmental expression of prestin coincides with the appearance of electromotility [4]. When prestin is expressed in non-auditory mammalian cells *in vitro*, a non-linear, voltage-dependent membrane capacitance, a commonly used marker of electromotility, results [3,5]. Targeted deletion of prestin in mice eliminates OHC electromotility and results in a significant (40-60dB) loss of cochlear sensitivity and an increased high frequency hearing threshold [3,6,7]. The human gene *SLC26A5*, encoding prestin, contains 21 exons that are alternately spliced to create four isoforms of prestin with variable lengths of the C-terminus [8]. The expression profile of the four isoforms in OHCs and their significance with respect to electromotility are not known [8].

In humans, a single nucleotide change in the second intron of *SLC26A5 *has been reported in association with hearing loss [8]. The DNA sequence variation, IVS2-2A>G, is an A to G transition in the splice acceptor site for exon 3. The A nucleotide at position IVS2-2 of *SLC26A5 *was found to be conserved in human, mouse, and rat orthologs and mutation of this nucleotide is predicted to cause skipping of exon 3 [8-10]. The start codon for the prestin protein is encoded in exon 3 of *SLC26A5 *[8,10]. Skipping of exon 3 during RNA processing would be expected to result in a messenger RNA incapable of prestin protein production and cochlear hearing loss due to absence of OHC electromotility [8-10].

To further explore the relationship between the *SLC26A5 *IVS2-2A>G nucleotide substitution and hearing loss, and assess allele frequency, genomic DNA from hearing impaired and control subjects was genotyped at the *SLC26A5 *IVS2-2 position. In addition, *SLC26A5 *orthologs from various eukaryotic species were evaluated for conservation of the A nucleotide at position IVS2-2. An attempt was also made to identify possible alternative splice acceptor sites within intron 2 that might support alternate splicing of exon 3.

## Methods

### Subjects

Hearing impaired patients were identified and recruited from the outpatient clinical care centers of the Bobby R. Alford Department of Otorhinolaryngology and Communicative Sciences and the Department of Molecular and Human Genetics of Baylor College of Medicine. Ethnicity of cases was self-described. Control specimens were obtained from the Baylor Polymorphism Resource . In this resource, Caucasian subjects were European Americans. Ethnicity of controls was recorded by the Baylor Polymorphism Resource group at the time of specimen collection. The hearing status of controls is unknown however the process through which control subjects were recruited included verbal communication, reducing the likelihood that individuals with significant hearing impairment were unknowingly included in the control population.

### IRB approval

This work was approved by the Baylor College of Medicine Institutional Review Board. Informed consent was obtained and documented from all subjects prior to specimen collection.

### Specimen collection

Blood was collected from all subjects by peripheral venipuncture. Lymphoblastoid cell lines were established by standard Epstein Barr Virus mediated transformation protocols.

### DNA isolation

DNA was isolated from cultured cells using the PUREGENE^® ^DNA Purification Kit for cells (Gentra Systems, Inc., Minneapolis, Minnesota, USA), according to manufacturer's specifications.

### Sequencing of the *SLC26A5 *exon 3 splice acceptor site

The region surrounding the exon 3 splice acceptor site of *SLC26A5 *was amplified from human genomic DNA by polymerase chain reaction (PCR) using the forward primer Prestin-5'F from [8] and, a reverse primer that was derived from primer Prestin-4R from [8] by the addition of 5 nucleotides to the 5' end as follows, 5'-GCAATTGTTTGAGGACAGCAAGGG-3'. PCR was conducted with 15 pmol of each primer, 1.25U Taq DNA polymerase (Amersham Pharmacia Biotech Inc., Piscataway, NJ, USA), and 1 × PCR buffer, as provided by the manufacturer, in a total volume of 25 μL. PCR was conducted as follows: 94°C for 2 minutes; 40 cycles of 94°C for 30 seconds, 58°C for 30 seconds, and 70°C for 1 minute; and, 70°C for 5 minutes. PCR fragments were sequenced using the forward primer and the ABI BigDye Terminator v3.1 Cycle Sequencing Kit (Applied Biosystems Inc, Foster City, California, USA). Cycle sequencing was conducted as follows: 25 cycles of 96°C for 10 seconds; 50°C for 5 seconds; and, 60°C for 4 minutes. Sequencing reactions were analyzed on an ABI Prism 310 Genetic Analyzer according to the manufacturer's specifications (Applied Biosystems, Foster City, CA).

### Sequencing of the coding regions of *GJB2 *(Connexin 26) and *GJB6 *(Connexin 30)

The coding region of *GJB2 *was amplified by PCR and sequenced using the following primers: forward PCR primer, 5'-AGGAAGAGATTTAAGCATGCT-3'; reverse PCR primer, 5'-TGGAGTTTCACCTGAGGC-3'; forward sequencing primer, 5'-CTGCAGCTGATCTTCGTG-3'; and, reverse sequencing primer, 5'-GTCGTACATGACATAGAAGACGT-3'. The coding region of *GJB6 *was amplified from human genomic DNA by PCR using the following primers: forward PCR primer derived from primer Cx30-1 from [11] by the addition of 3 nucleotides to the 5' end and the removal of 2 nucleotides from the 3' end as follows, 5'-GACTCAGGGATAAACCAGCGCA-3'; reverse PCR and sequencing primer Cx30-8 from [11], and reverse sequencing primer Cx30-4 from [11]. PCR and sequencing were conducted as described for the *SLC26A5 *exon 3 splice acceptor site.

### *GJB6 *(Connexin 30) deletion analysis

The *GJB6 *gene was evaluated for the 342 kilobase pair deletion shown to be associated with hearing loss as described [11-13].

### Mitochondrial 12S rRNA gene sequencing and restriction analysis for the 1555A>G mutation

DNA samples were amplified by PCR using primers derived from MITOMAP and PCR conditions as previously described [14,15]. The presence of the 1555A>G mutation was evaluated in some subjects by DNA sequencing as previously described [15] and in other subjects by restriction digestion using BsmAI (New England BioLabs, Beverly, MA, USA) according to manufacturer's specifications and as described [16].

### Sequence analysis

Electropherograms were evaluated by visual inspection and pairwise alignment to reference sequences using the BCM Search Launcher BLAST2 Pairwise Sequence Alignment Tool from the Human Genome Sequencing Center of Baylor College of Medicine , and/or by interpretation using Mutation Surveyor software Version 2.41 (Softgenetics, Inc, State College, Pennsylvania, USA).

### Statistical analysis

Fisher's exact test of 2 × 2 contingency tables was used to calculate the two-tailed p values associated with allele frequencies in case and control groups. Fisher's exact test was performed using the GraphPad QuickCalcs Online Calculator for Scientists .

### Multiple sequence alignment of the exon 3 splice acceptor site of *SLC26A5*

*SLC26A5 *genomic DNA sequences from human, chimp, rat, mouse, zebrafish and fruit fly were obtained from the Ensembl Genome Browser . Prestin sequences at the intron 2 and exon 3 junction were compared using ClustalW multiple sequence alignment tool, Biology WorkBench 3.2 .

### Evaluation of potential alternative splice acceptor sites within intron 2 of human *SLC26A5*

Alternative splice acceptor sites within intron 2 of human *SLC26A5 *were sought using a splice site prediction program from the Berkeley Drosophila Genome Project . Only sites having a confidence score above 0.99 out of a maximum possible score of 1.00 were considered to be potential alternative splice sites.

## Results

### Allele frequency of the IVS2-2A>G DNA sequence variation

Eighty-four hearing impaired cases and 246 controls were genotyped for the exon 3 splice acceptor site of *SLC26A5*, encoding prestin. Four hearing impaired cases were found to be heterozygous for the IVS2-2A>G nucleotide substitution: one Hispanic, two Caucasian, and one of uncertain ethnicity, possibly Caucasian or mixed Caucasian. Four controls were also found to be heterozygous for the IVS2-2A>G nucleotide substitution: one Hispanic and three Caucasian (Table 1). Four of the eight carriers were male: three cases and one control. Four of the eight carriers were female: three controls and one case. The IVS2-2A>G nucleotide substitution was not found in 106 subjects of Asian or African American descent nor was it found in a homozygous state in any of 330 total subjects (Table 1).

Data from the control population suggests an allele frequency for the IVS2-2A>G nucleotide substitution of approximately 0.007 in Hispanics (carrier frequency 1.3%) and 0.02 in European American Caucasians (carrier frequency 4%). The difference in allele frequency of the IVS2-2A>G nucleotide substitution between cases and controls was evaluated by derivation of two-tailed p values using Fisher's exact test of 2 × 2 contingency tables. The difference in allele frequency of the IVS2-2A>G variant between cases and controls among Hispanics and Caucasians in this population was not statistically significant (p = 0.45). If Asian and African American cases and controls are included in the Fisher's exact test analysis, the allele frequency distribution still does not reach statistical significance (p = 0.12, Table 1).

The phenotype and audiometric profile of each of the four hearing impaired subjects who were carriers of the *SLC26A5 *IVS2-2A>G nucleotide substitution was unique (Table 2). Of note, the hearing impaired sibling of case #4 did not carry the IVS2-2A>G DNA sequence variation. Among the four *SLC26A5 *IVS2-2A>G carriers, hearing loss associated mutations were not found in the coding regions of *GJB2 *or *GJB6*, encoding Connexin 26 and Connexin 30 respectively. The deletion mutation in *GJB6 *[11-13] and the 1555A>G mutation in the mitochondrial 12S rRNA gene [16-18] were also absent from these hearing impaired *SLC26A5 *IVS2-2A>G carriers. Neither genetic nor hearing tests were performed on the parents or other relatives of these subjects.

### Multiple sequence alignment of the exon 3 splice acceptor site of *SLC26A5*

*SLC26A5 *genomic DNA sequences from human, chimp, rat, mouse, zebrafish and fruit fly were aligned and compared for evolutionary conservation of the exon 3 splice acceptor site and, in particular, the AG dinucleotide. The A and G nucleotides at positions -2 and -1, respectively, of the exon 3 splice acceptor site were invariably present and evolutionarily conserved among all eukaryotic prestin orthologs analyzed (Table 3).

### Evaluation of alternative splice sites within intron 2 of *SLC26A5*

**Figure 1:**
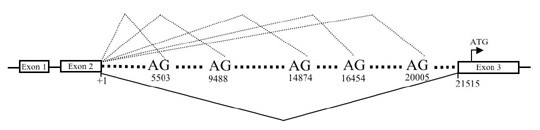
**Potential alternate splicing of intron 2 of *SLC26A5 *containing the IVS2-2A>G variant. **Alternative splice acceptor sites in intron 2 of *SLC26A5 *having a confidence score above 0.99 out of a maximum possible score of 1.00 are shown. Numbering of intron 2 begins at the +1 position. Dotted lines above the gene drawing show potential alternately spliced gene products. The solid line below the gene drawing shows the wild type splice donor and acceptor sites that join exon 2 to exon 3.

To identify possible alternative splice acceptor sites within intron 2 of *SLC26A5 *that might support alternate splicing of exon 3, the 21,515 base pair genomic DNA sequence of intron 2 was analyzed using a splice site prediction program from the Berkeley Drosophila Genome Project . Five alternative splice acceptor sites demonstrating a confidence score greater than 0.99 out of a maximum of 1.00 were identified within intron 2 of *SLC26A5 *(Figure 1).

## Discussion

These data suggest that heterozygosity for the *SLC26A5 *IVS2-2A>G DNA sequence variation may not be, by itself, sufficient to cause hearing loss. Only one of the four hearing impaired carriers of the *SLC26A5 *IVS2-2A>G DNA sequence variation identified in this study reported a history of hearing loss in a parent. Further, various additional clinical findings in each of the hearing impaired carriers of the *SLC26A5 *IVS2-2A>G variant argue against *SLC26A5 *being the cause of hearing loss in each of these cases (Table 2).

The carrier frequency for the *SLC26A5 *IVS2-2A>G DNA sequence variation in the control populations used in this study was observed to be 1.3% in Hispanics and 4% in Caucasians. These data suggest that the *SLC26A5 *IVS2-2A>G DNA sequence variation may not be uncommon and that it occurs in multiple ethnic groups, in contrast to previous studies [8]. The *SLC26A5 *IVS2-2A>G DNA sequence variation was not found in Asians or African Americans. This observation may reflect absence of the variant in these ethnic groups or may be due to the small size of the study population.

Although nothing is known about the hearing status of the control group, the allele frequency of the *SLC26A5 *IVS2-2A>G variant was not significantly different between cases and controls. In addition, the *SLC26A5 *IVS2-2A>G variant was not found in homozygosity in any hearing impaired case. Given the high allele frequency of the IVS2-2A>G variant in the Caucasian and Hispanic control populations, if this variant were associated with hearing loss, it is reasonable to expect that homozygous hearing impaired cases would have been observed in the study group. This expectation is based on the following observations. First, congenital genetic hearing loss affects, conservatively, approximately 1 in 2,000 live births in the United States [19]. A carrier rate in Caucasians of 4% for the *SLC26A5 *IVS2-2A>G nucleotide substitution suggests that approximately 1 in 2,500 Caucasians should be homozygous for this mutation. If this DNA sequence variation were related to hearing loss, it should account for a large percentage of cases of early onset genetic hearing loss in Caucasians. Curiously, *SLC26A5 *has not been mapped by traditional linkage analysis in any of the families with hereditary hearing loss that have so far been studied worldwide (Van Camp G, Smith RJH. Hereditary Hearing Loss Homepage. . Further, the carrier rate among Caucasians for mutations in *GJB2*, encoding Connexin 26, widely believed to be the most common cause of early onset genetic hearing loss in the United States, is estimated to be approximately 3% [20]. This is 1% less than the carrier frequency of the *SLC26A5 *IVS2-2A>G DNA sequence variation observed among Caucasians in this study. The common 35delG mutation in *GJB2 *is, by itself, estimated to have a carrier rate in US Caucasians of approximately 2.5% [20]. In a group of 94 hearing impaired cases that includes the 84 subjects tested in this study for *SLC26A5 *IVS2-2A>G, five subjects homozygous for the 35delG mutation were found (unpublished observation). In contrast, no subjects homozygous for the IVS2-2A>G DNA sequence variation were observed. These observations challenge whether the *SLC26A5 *IVS2-2A>G DNA sequence variation is associated with hearing loss.

As shown in Table 3, the AG dinucleotide at the splice acceptor site of exon 3 of *SLC26A5 *is invariant and evolutionarily conserved amongst prestin orthologs. Analysis of gene structure in multiple species including vertebrates, invertebrates, plants and viruses suggested that the AG dinucleotide of 3' splice acceptor sites cannot tolerate mutation [9]. Thus, the IVS2-2A>G substitution creates a sequence variation in *SLC26A5 *that is expected to cause skipping of exon 3 [8,9]. However, since a number of potential alternative splice acceptor sites exist within intron 2 of human *SLC26A5 *(Figure 1), and the translation start codon for prestin is found within exon 3 [8,10], alternate splicing of exon 3 within intron 2 might be compatible with production of a correctly translated prestin protein. Alternatively, utilization of one or more alternate splice sites in a way that minimizes the number of transcripts missing exon 3 during functional maturation of the cochlea might minimally impact the level of prestin in mature OHCs. This hypothesis requires *in vivo *evaluation of splicing and steady state protein levels in cells carrying *SLC26A5 *genes with the IVS2-2A>G substitution, and in particular during functional maturation of the cochlea.

Further studies are needed to clarify and refine the IVS2-2A/G allele frequencies in various ethnic groups and examine the role of the IVS2-2A>G nucleotide substitution in hearing loss. These studies should include screening of additional case and control populations for the *SLC26A5 *IVS2-2A>G DNA sequence variation, and identification and audiometric testing of carriers and homozygous individuals.

## Conclusion

The exon 3 splice acceptor sequence of *SLC26A5 *is evolutionarily conserved. Disruption of the canonical AG dinucleotide at this splice acceptor site by the IVS2-2A>G transition is expected to abolish splice site recognition and result in skipping of exon 3. The IVS2-2A>G transition is found in individuals of Hispanic and European American (Caucasian) ancestry, suggesting this DNA sequence variation is not limited to a single ethnic group. Among Hispanics and Caucasians, there is no statistically significant difference in allele frequency of the IVS2-2A>G nucleotide substitution between hearing impaired cases and controls, challenging whether this DNA sequence variation is associated with hearing loss. The detection of alternative splice acceptor sites within intron 2 suggests a potential mechanism by which expression of prestin might be maintained in cells carrying the *SLC26A5 *IVS2-2A>G DNA sequence variation. Further studies of the *SLC26A5 *IVS2-2A>G DNA sequence variation are needed.

## Competing interests

The author(s) declare that they have no competing interests.

## Authors' contributions

HYT participated in the design of this study, carried out the laboratory analyses, contributed to the analysis and interpretation of the data, and helped draft the manuscript. APX participated in the analysis and interpretation of the molecular genetic data, performed the sequence alignment and alternative splice site analyses, and helped to draft the manuscript. JSO participated in the design of this study, in the analysis and interpretation of the data, and in preparation of the manuscript. FAP participated in the design of the study, the sequence alignment and alternative splice site analyses, the analysis and interpretation of the data, and preparation of the manuscript. RLA participated in the design of the study, was responsible for subject enrollment and control group acquisition, analyzed and interpreted data from the study, and prepared the manuscript. All authors read and approved of the final manuscript.

## Pre-publication history

The pre-publication history for this paper can be accessed here:



## Figures and Tables

**Table 1:** Genotypes of cases and controls at position *SLC26A5 *IVS2-2.

Ethnicity	Case Genotypes		Control Genotypes		Fisher's exact test two-tailed p value	
	A/A	A/G	A/A	A/G		

Hispanic	22	1	75	1	p = 0.41	p = 0.45
Caucasian	34	2	71	3	p = 0.66	
Uncertain (Caucasian/Mixed Caucasian)	14	1	0	0	Not Done	
Asian	3	0	50	0	Not Done	
African American	7	0	46	0	Not Done	
Total	80	4	242	4	p = 0.12	

**Table 2:** Phenotypes of hearing impaired carriers of *SLC26A5 *IVS2-2A>G.

Case	Type of Hearing Loss; Age at onset	Associated Anomalies	Family History	Ethnicity	*GJB2*, *GJB6, 12S rRNA*
1	Profound sensorineural on right, severe conductive on left; Congenital	Internal auditory canal hypoplasia, hypoplastic vestibulocochlear nerve on right; External auditory canal atresia, malformed ossicles on left	None reported	Hispanic	Heterozygous *GJB2 *V27I polymorphism, no mutations or other variants found
2	Severe to profound on right, moderate to severe on left, mixed, progressive; Early childhood, sudden, with head trauma and ear infection	Bilateral enlarged vestibular aqueducts; Disequilibrium; History of ear infections and possible meningitis	None reported	Caucasian	No mutations or variants found
3	Moderate to severe, bilateral, sloping audiogram, sensorineural, progressive; Childhood to young adulthood	None	Consistent with autosomal dominant inheritance	Caucasian	No mutations or variants found
4	Moderate on right, mild to moderate on left, sensorineural, progressive; Childhood	None	Hearing impaired sibling (progressive, mild on right, mild to moderate on left, sensorineural; childhood onset; renal malformation); hearing impaired grandparent (unknown severity, possibly noise-induced)	Uncertain: Caucasian/Mixed Caucasian	No mutations or variants found

**Table 3:** Multiple sequence alignment of the exon 3 splice acceptor site of *SLC26A5*.

**Species**	Intron 2/EXON 3*	**Ensembl Gene ID**
Human	cccct**ag**/TGACACT	ENSG00000170615
Chimp	cccct**ag**/TGACACT	ENSPTRG00000019554
Rat	cctgc**ag**/GCTTAGC	ENSRNOG00000011616
Mouse	ccctt**ag**/TGGCCAT	ENSMUSG00000029015
Zebrafish	ttttc**ag**/CTGTTCG	ENSDARG00000022424
Fruit fly	ttttc**ag**/CTCCTAA	CG5485

*The invariant AG dinucleotide at the intron 2/exon 3 splice acceptor site is shown in bold and underlined.
